# Effect of Chewing Gum on Postoperative Ileus Following Surgery for Gastroduodenal Perforation: A Prospective Non-randomized Comparative Study

**DOI:** 10.7759/cureus.110577

**Published:** 2026-06-10

**Authors:** Bindu N, Sreeramulu PN, Prakash Dave

**Affiliations:** 1 General Surgery, Sri Devaraj Urs Academy of Higher Education and Research (SDUAHER), Kolar, IND; 2 Surgery, RL Jalappa Hospital and Research Centre, Sri Devaraj Urs Academy of Higher Education and Research (SDUAHER), Kolar, IND; 3 General Surgery, Sri Devaraj Urs Medical College, Kolar, IND

**Keywords:** bowel motility, chewing gum, emergency laparotomy, gastroduodenal perforation, gastrointestinal recovery, general surgery, perforation peritonitis, postoperative ileus, prospective comparative study, sham feeding

## Abstract

Background

Postoperative ileus (POI) is a common complication following emergency laparotomy for gastroduodenal perforation and is associated with delayed recovery, prolonged hospital stay, and increased postoperative morbidity. Chewing gum acts as a form of sham feeding and may stimulate gastrointestinal motility through cephalic-vagal stimulation. Although its benefits have been demonstrated in elective abdominal surgeries, evidence regarding its role in emergency perforation surgeries remains limited.

Methods

This prospective comparative study was conducted in the Department of General Surgery at Sri Devaraj Urs Medical College and Hospital, Kolar, between April 2025 and October 2025. Fifty patients undergoing emergency laparotomy for gastroduodenal perforation were included in the study and divided into two groups of 25 patients each using an odd-even allocation method. Patients in the intervention group received sugar-free chewing gum for 20 minutes three times daily beginning six hours after surgery until the first passage of flatus, whereas the control group received standard postoperative care alone. The primary outcome measure was time to first passage of flatus. Secondary outcomes included time to first stool, incidence of nasogastric tube reinsertion, duration of hospital stay, and postoperative complications.

Results

The mean time to first passage of flatus was significantly lower in the chewing gum group compared with the control group (32.6 ± 6.4 hours vs. 45.8 ± 8.1 hours, p < 0.001). Patients in the chewing gum group also demonstrated earlier passage of stool and shorter duration of hospital stay. The incidence of postoperative complications was lower in the chewing gum group, although the difference was not statistically significant.

Conclusions

Postoperative chewing gum therapy significantly improved recovery of bowel function following emergency laparotomy for gastroduodenal perforation. Chewing gum is a simple, safe, inexpensive, and non-pharmacological intervention that may reduce POI and enhance postoperative recovery in emergency abdominal surgeries.

## Introduction

Gastroduodenal perforation is one of the most common surgical emergencies encountered in developing countries and is associated with significant postoperative morbidity and prolonged hospital stay [1,2]. Emergency laparotomy with simple closure of the perforation, with or without an omental (Graham) patch, remains the standard surgical treatment modality; however, postoperative ileus (POI) continues to be a frequent complication following abdominal surgery, particularly in cases involving bowel manipulation and peritoneal contamination [3,4].

POI is defined as a transient impairment of gastrointestinal motility following surgery in the absence of mechanical bowel obstruction. Although a brief period of gastrointestinal dysmotility is expected after abdominal surgery, persistence of symptoms such as abdominal distension, nausea, vomiting, intolerance to oral intake, and delayed passage of flatus or stool beyond the first 24-72 hours is generally considered indicative of POI [3]. Clinically, it manifests as abdominal distension, delayed passage of flatus and stool, nausea, vomiting, intolerance to oral feeds, and prolonged nasogastric decompression [3]. The pathophysiology of POI is multifactorial and involves bowel handling during surgery, inflammatory mediator release, electrolyte imbalance, sympathetic neural inhibition, and opioid analgesic use [4].

The incidence of POI following abdominal surgery has been reported to be as high as 30%, particularly in patients undergoing emergency procedures, extensive bowel manipulation, or surgery in the presence of intra-abdominal sepsis and peritonitis [3,4]. Therefore, strategies aimed at accelerating recovery of bowel function are essential in improving postoperative outcomes.

Gastroduodenal perforation resulting in perforation peritonitis is a life-threatening surgical emergency associated with substantial morbidity and mortality, particularly in developing countries. Reported mortality rates range from approximately 6% to 27%, depending on patient age, comorbidities, extent of peritoneal contamination, and delay in presentation. Patients with perforation peritonitis are at increased risk of postoperative complications, including POI, owing to intra-abdominal sepsis, bowel edema, inflammatory response, and extensive bowel manipulation during surgery. Consequently, delayed gastrointestinal recovery remains an important contributor to prolonged hospitalization and postoperative morbidity in this patient population.

Chewing gum has emerged as a simple, inexpensive, and non-pharmacological sham feeding intervention for enhancing postoperative gastrointestinal recovery. It is increasingly recognized as an Enhanced Recovery After Surgery (ERAS)-aligned strategy that may facilitate earlier return of bowel function while avoiding additional pharmacological interventions [5,6]. This mechanism promotes intestinal motility through increased salivary and pancreatic secretions, vagal stimulation, and release of gastrointestinal hormones such as gastrin and neurotensin [5,6].

Several randomized controlled trials conducted in elective colorectal and gynecological surgeries have demonstrated earlier return of bowel function and reduced duration of POI with chewing gum therapy [7-9]. Asao et al. were among the first to demonstrate that chewing gum significantly shortened the time to first passage of flatus and bowel movement following laparotomy [1]. Subsequently, Chan and Law, Quah et al., and Schuster et al. reported improved postoperative gastrointestinal recovery and shorter hospital stay in patients receiving chewing gum therapy after colorectal surgeries [7-9].

A systematic review and meta-analysis by Noble et al. demonstrated that chewing gum significantly reduced POI and shortened the duration of hospital stay following gastrointestinal surgery [2]. Similarly, Fitzgerald and Ahmed and Li et al. confirmed the beneficial effects of chewing gum therapy on postoperative bowel recovery across multiple randomized controlled trials [5,6].

Despite these encouraging findings, most available studies have been conducted in elective surgical populations. Emergency laparotomy for gastroduodenal perforation involves severe peritoneal contamination, bowel inflammation, and increased physiological stress, which may influence postoperative gastrointestinal recovery differently compared with elective surgeries [4]. Evidence regarding the effectiveness of chewing gum therapy in this subgroup remains limited, particularly in developing healthcare settings where perforation peritonitis continues to be highly prevalent [10]. Therefore, the present study was conducted to evaluate the effect of postoperative chewing gum therapy on the duration of POI following emergency laparotomy for gastroduodenal perforation, with particular emphasis on time to first passage of flatus as a marker of gastrointestinal recovery.

## Materials and methods

Study design and setting

This prospective comparative study was conducted in the Department of General Surgery at Sri Devaraj Urs Medical College and Hospital, Tamaka, Kolar, Karnataka, India, between April 2025 and October 2025 after obtaining approval from the Central Ethics Committee of Sri Devaraj Urs Academy of Higher Education and Research. The study was designed to evaluate the effect of postoperative chewing gum therapy on the duration of POI following emergency laparotomy for gastroduodenal perforation [5,6].

Study population

Consecutive patients diagnosed with gastroduodenal perforation based on clinical evaluation and radiological findings and undergoing emergency laparotomy during the study period were screened for eligibility. Patients fulfilling the inclusion criteria and willing to provide written informed consent were included in the study.

Inclusion and exclusion criteria

Patients aged more than 18 years undergoing emergency laparotomy for gastroduodenal perforation were included in the study. Patients with previous major abdominal surgery, those requiring bowel resection or stoma formation, patients requiring prolonged postoperative ventilatory support, patients with neurological or psychiatric disorders affecting chewing or swallowing, and patients with oral cavity disorders preventing chewing gum use were excluded from the study [3,4].

Sample size

The sample size was calculated based on previous studies evaluating the effect of chewing gum therapy on postoperative gastrointestinal recovery following abdominal surgery [2,5,6]. Assuming a mean difference of approximately 12 hours in time to first passage of flatus between the intervention and control groups, with a pooled standard deviation (SD) of 14 hours, a two-sided confidence level of 95% (α = 0.05), and a study power of 80% (β = 0.20), the minimum required sample size was calculated to be 22 patients per group using the formula for comparison of two independent means:

$n = 2\times (Z_{\alpha/2} + Z_{\beta})^{2}\times \sigma^{2} / d^{2}$

where n is the sample size per group, Z_α/2_ = 1.96 for a 95% confidence interval, Z_β_ = 0.84 for 80% power, σ is the pooled SD, and d is the expected difference between the two groups.

To account for possible exclusions, protocol deviations, and loss to follow-up, the sample size was increased to 25 patients in each group, resulting in a total sample size of 50 patients. The sample size calculation was based on previously published studies evaluating chewing gum therapy and postoperative gastrointestinal recovery.

Patient allocation

Patients were allocated to either the chewing gum group or the control group using an odd-even allocation method based on the order of recruitment. Patients assigned odd serial numbers were included in Group A (chewing gum group), while patients assigned even serial numbers were included in Group B (control group). Although this method ensured balanced allocation between groups, it does not constitute true randomization.

Intervention protocol

Patients in Group A received commercially available sugar-free chewing gum approximately six hours after surgery once they were fully conscious and able to chew safely [1,5]. Patients were instructed to chew gum for 20 minutes per session, three times daily, until the first passage of flatus. Compliance with chewing gum therapy was monitored and documented by the nursing staff in postoperative records. Patients in Group B received standard postoperative care alone.

Postoperative management

All patients in both groups received standard postoperative care according to institutional protocol, including nasogastric decompression when required, intravenous fluids and electrolyte correction, broad-spectrum antibiotics, analgesia, early ambulation, and regular monitoring of vital parameters. Postoperative nutritional management was standardized for both groups. Oral sips of water were initiated once patients demonstrated clinical evidence of gastrointestinal recovery and were deemed suitable for enteral intake by the treating surgical team [10]. This was followed by progression to clear liquids and subsequently a soft oral diet as tolerated. The same postoperative feeding protocol was followed in both groups to minimize the influence of nutritional management on gastrointestinal recovery outcomes.

Postoperative analgesia was administered according to institutional protocols and consisted of standard analgesic therapy as determined by the treating surgical team. Early ambulation was encouraged in all patients. No additional sham feeding interventions or specific bowel stimulation strategies were employed during the study period. Apart from the chewing gum intervention, postoperative management was comparable between the two groups.

Outcome measures

The primary outcome measure was time to first passage of flatus following surgery. Secondary outcome measures included time to first stool, incidence of nasogastric tube reinsertion, duration of hospital stay, and postoperative complications [2,6].

Data collection

Patient demographic details, clinical presentation, operative findings, postoperative recovery parameters, and postoperative complications were recorded using a predesigned proforma. Patients were followed until discharge from the hospital.

Statistical analysis

Data were entered into Microsoft Excel (Microsoft Corp., Redmond, WA, USA) and analyzed using Statistical Package for the Social Sciences (SPSS) software version 22.0 (IBM Corp., Armonk, NY, USA). Continuous variables such as time to first passage of flatus, time to first stool, and duration of hospital stay were expressed as mean ± SD. Categorical variables such as sex distribution, perforation site, nasogastric tube reinsertion, and postoperative complications were expressed as frequencies and percentages.

The normality of data distribution was assessed using the Shapiro-Wilk test. Normally distributed continuous variables were compared using the independent Student’s t-test, whereas non-normally distributed variables were analyzed using the Mann-Whitney U test. Categorical variables were analyzed using the Chi-squared test or Fisher’s exact test wherever appropriate. A p-value of less than 0.05 was considered statistically significant [11,12].

Ethical considerations

Ethical approval for the study was obtained from the Central Ethics Committee of Sri Devaraj Urs Academy of Higher Education and Research, Kolar. Written informed consent was obtained from all participants prior to inclusion in the study. Patient confidentiality was strictly maintained throughout the study.

## Results

A total of 50 patients undergoing emergency laparotomy for gastroduodenal perforation were included in the study. Patients were allocated into two groups using the odd-even method of allocation, with 25 patients each in the chewing gum group (Group A) and the standard postoperative care group (Group B). All enrolled patients completed the study and were included in the final analysis. No patient required re-exploration during the postoperative period, and therefore, no participant was excluded from the final analysis after allocation.

Baseline demographic and clinical characteristics of the study participants are summarized in Table 1. There was no statistically significant difference between the two groups with respect to age, sex distribution, perforation site, duration of symptoms, or duration of surgery (p > 0.05).

**Table 1:** Baseline Demographic and Clinical Characteristics of Study Participants Independent Student’s t-test was applied for comparison of continuous variables including age, duration of symptoms before surgery, and duration of surgery. The Chi-squared test was applied for categorical variables including gender distribution, gastric perforation, and duodenal perforation. SD: standard deviation

Variables	Group A (chewing gum) (n = 25)	Group B (control) (n = 25)	Test statistic	p-value
Age (years), mean ± SD	44.8 ± 11.2	46.3 ± 10.5	t = 0.51	0.61
Gender (male/female)	21/4	20/5	χ² = 0.12	0.71
Gastric perforation, n (%)	9 (36%)	8 (32%)	χ² = 0.09	0.76
Duodenal perforation, n (%)	16 (64%)	17 (68%)	χ² = 0.09	0.76
Duration of symptoms before surgery (hours), mean ± SD	18.4 ± 5.3	19.1 ± 4.9	t = 0.48	0.63
Duration of surgery (minutes), mean ± SD	94.2 ± 14.1	96.5 ± 13.6	t = 0.61	0.54

Comparison of the primary outcome between the two groups is shown in Table 2. The mean time to first passage of flatus was significantly lower in the chewing gum group compared with the control group (32.6 ± 6.4 hours vs. 45.8 ± 8.1 hours, p < 0.001).

**Table 2:** Comparison of Primary Outcome Between the Two Groups Independent Student’s t-test was applied for comparison of time to first passage of flatus between the two groups. SD: standard deviation

Primary outcome	Group A (n = 25)	Group B (n = 25)	Test statistic	p-value
Time to first passage of flatus (hours), mean ± SD	32.6 ± 6.4	45.8 ± 8.1	t = 6.45	<0.001

Secondary postoperative outcomes between the two groups are summarized in Table 3. Patients in the chewing gum group demonstrated earlier return of bowel function and shorter duration of hospital stay compared with the control group. The incidence of nasogastric tube reinsertion and postoperative complications was lower in the chewing gum group, although the differences were not statistically significant.

**Table 3:** Comparison of Secondary Outcomes Between the Two Groups Independent Student’s t-test was applied for continuous variables including time to first stool and duration of hospital stay. The Chi-squared test was applied for categorical variables including nasogastric tube reinsertion and postoperative complications. SD: standard deviation

Secondary outcomes	Group A (n = 25)	Group B (n = 25)	Test statistic	p-value
Time to first stool (hours), mean ± SD	49.2 ± 8.7	63.4 ± 10.2	t = 5.32	<0.001
Nasogastric tube reinsertion, n (%)	2 (8%)	5 (20%)	χ² = 1.42	0.22
Duration of hospital stay (days), mean ± SD	6.4 ± 1.5	8.1 ± 1.9	t = 3.41	0.002
Postoperative complications, n (%)	4 (16%)	7 (28%)	χ² = 1.08	0.18

Postoperative complications observed in both groups are presented in Table 4. Surgical site infection was the most common postoperative complication observed in the study. No adverse effects directly related to chewing gum therapy were noted during the study period.

**Table 4:** Postoperative Complications Observed in Both Groups The Chi-squared test was applied for comparison of surgical site infection, fever, prolonged ileus, and total postoperative complications between the two groups. Fisher’s exact test was applied for respiratory complications because of small expected cell counts.

Complications	Group A (n = 25)	Group B (n = 25)	Test statistic	p-value
Surgical site infection	2	3	χ² = 0.22	0.63
Fever	1	2	χ² = 0.36	0.55
Prolonged ileus	1	1	χ² = 0.00	1.00
Respiratory complications	0	1	Fisher’s exact test	0.31
Total complications	4	7	χ² = 1.08	0.18

## Discussion

The present study demonstrated that postoperative chewing gum significantly improved recovery of bowel function following emergency laparotomy for gastroduodenal perforation. Patients receiving chewing gum therapy experienced earlier passage of flatus and stool, as well as a shorter duration of hospital stay, compared with patients receiving standard postoperative care alone.

POI remains one of the most common complications following abdominal surgery and contributes significantly to delayed recovery, prolonged hospital stay, increased healthcare expenditure, and patient discomfort [3,11,13]. Emergency laparotomy for gastroduodenal perforation is associated with a particularly high risk of POI because of bowel manipulation, peritoneal contamination, inflammatory response, and postoperative opioid use [4,14]. Various strategies have been investigated to enhance postoperative gastrointestinal recovery, among which chewing gum has emerged as a simple, inexpensive, and non-pharmacological intervention.

The present study demonstrated that postoperative chewing gum therapy significantly reduced the time to first passage of flatus compared with standard postoperative care alone. Patients in the chewing gum group also demonstrated earlier passage of stool and shorter duration of hospital stay. These findings suggest that sham feeding through chewing gum stimulates gastrointestinal motility and accelerates postoperative bowel recovery.

The beneficial effects of chewing gum are thought to be mediated through sham feeding mechanisms that stimulate cephalic-vagal pathways and enhance gastrointestinal motility. Previous physiological studies have suggested that chewing gum may increase the release of gastrointestinal hormones such as gastrin and neurotensin, thereby promoting bowel activity and accelerating recovery of gastrointestinal function [5,6,12]. However, these hormonal effects were not directly measured in the present study and are discussed as proposed mechanisms based on prior physiological and clinical research rather than findings from our study cohort [5,12].

The findings of the present study are consistent with those reported by Asao et al., who were among the first to demonstrate that gum chewing after laparotomy significantly shortened the time to first passage of flatus and bowel movement [1]. Similarly, Schuster et al. reported earlier recovery of bowel function and reduced POI in patients receiving chewing gum therapy after colorectal surgery [9].

Several randomized controlled trials and meta-analyses have further supported the beneficial effects of chewing gum therapy following abdominal surgery. Noble et al. demonstrated that chewing gum significantly reduced time to first flatus, first bowel movement, and duration of hospital stay [2]. Fitzgerald and Ahmed also reported significant improvement in postoperative gastrointestinal recovery with chewing gum therapy following gastrointestinal surgeries [5]. Another meta-analysis by Li et al. demonstrated improved postoperative bowel recovery and reduced duration of POI across multiple randomized controlled trials [6].

In the current study, the incidence of nasogastric tube reinsertion and postoperative complications was lower in the chewing gum group, although the difference did not reach statistical significance. Similar findings have been reported in previous studies where chewing gum therapy improved bowel recovery without increasing postoperative complications [12,15]. Importantly, no adverse effects related to chewing gum therapy were observed during the study period, supporting its safety and feasibility in postoperative patients.

The shorter duration of hospital stay observed in the chewing gum group may have important clinical and economic implications, particularly in resource-limited healthcare settings. Early recovery of bowel function facilitates earlier oral feeding, mobilization, and discharge, thereby improving overall postoperative recovery and reducing healthcare burden [10,13].

Despite these encouraging findings, the present study has certain limitations. The present study has several limitations. First, the sample size was relatively small, which may have limited the ability to detect differences in less common postoperative outcomes and complications. Second, the study was conducted at a single tertiary care center, which may limit the generalizability of the findings to other healthcare settings and patient populations. Third, patient allocation was performed using a systematic odd-even allocation method rather than true randomization, introducing the possibility of selection bias. Fourth, neither patients nor investigators were blinded to the intervention, which may have resulted in performance and observer bias. In addition, a detailed assessment of compliance with chewing gum use and patient-reported outcomes such as comfort and satisfaction was not performed. Larger multicenter randomized controlled trials with blinded outcome assessment are therefore required to validate the present findings and establish standardized protocols for postoperative chewing gum use in emergency abdominal surgery.

An additional limitation is that the specific brand, flavor, and pellet size of the commercially available sugar-free chewing gum were not standardized or prospectively recorded as part of the study protocol. As these factors may influence salivary stimulation and patient acceptability, the exact reproducibility of the intervention may be limited.

Nevertheless, the present study demonstrated that chewing gum is a simple, safe, inexpensive, and easily implementable intervention that may significantly reduce POI following emergency laparotomy for gastroduodenal perforation.

## Conclusions

Postoperative chewing gum was associated with significantly earlier recovery of bowel function following emergency laparotomy for gastroduodenal perforation. Patients receiving chewing gum experienced passage of flatus approximately 13 hours earlier than those receiving standard postoperative care alone. Chewing gum also contributed to the earlier passage of stool and a shorter duration of hospital stay. Given its simplicity, low cost, and safety profile, chewing gum may serve as a useful adjunct to postoperative care in patients undergoing surgery for gastroduodenal perforation.

Chewing gum is a simple, safe, inexpensive, and non-pharmacological intervention that can be easily incorporated into routine postoperative management protocols. These findings are consistent with the hypothesis that chewing gum may enhance gastrointestinal recovery through stimulation of cephalic-vagal pathways and the release of gastrointestinal hormones such as gastrin and neurotensin, as reported in previous physiological studies. However, gastrointestinal motility and hormone levels were not directly measured in the present study. Further large-scale multicenter randomized controlled trials are recommended to validate these findings and establish standardized protocols for postoperative chewing gum therapy in surgical practice.
